# Dosage‐dependent effects of monensin on the rumen microbiota of lactating dairy cattle

**DOI:** 10.1002/mbo3.783

**Published:** 2018-12-18

**Authors:** Jeffery A. McGarvey, Sara Place, Jeffrey Palumbo, Robert Hnasko, Frank Mitloehner

**Affiliations:** ^1^ United States Department of Agriculture Agricultural Research Service Albany California; ^2^ National Cattlemen’s Association Sustainable Beef Production Research Centennial Colorado; ^3^ Department of Animal Science University of California Davis California

**Keywords:** 16S rRNA, dairy cattle, microbiota, monensin, Rumen

## Abstract

We examined the dose‐dependent effects of feeding lactating dairy cows a standard diet supplemented with monensin at 175, 368, or 518 mg cow^‐1^ day^‐1^on the rumen microbiota. For each dosage, 3 animals were randomly assigned into groups and fed the same basal total mixed ration diet supplemented with monensin, at the respective dose. After 20 days, rumen samples were taken and the effect on the microbiota was examined by 16S rRNA gene sequence analysis and qPCR. At the lowest dose no significant change in 16S rRNA gene sequences associated with any bacterial phyla was observed; however, at the medium and high dosages, we observed significant reductions in sequences associated with gram‐positive bacteria and significant increases in those associated with gram‐negative bacteria that were dosage dependent. All dosages reduced the levels of sequences associated with methanogenic archaea in the rumen, with the medium dosage showing the largest decline. No significant difference was observed for the 18S rRNA gene sequences associated with protozoa in any of the libraries. Our results indicate that with this diet the medium dosage of monensin was most efficacious for the reduction in methanogenic archaea in the rumen of lactating dairy.

## 1 INTRODUCTION

Monensin is a carboxylic polyether ionophore antibiotic (Haney & Hoehn, 1968) produced by *Streptomyces cinnamonensis* that was originally developed as a coccidiostat for chickens (Russell & Strobel, 1989). However, in the 1970s studies showed that beef cattle fed monensin had greater feed efficiency due to alterations in ruminal fermentation (Dinius, Simpson, & Marsh, 1976; Richardson, Raun, Potter, Cooley, & Rathmacher, 1976). Specifically, cattle fed monensin produced greater amounts of propionate that can be converted to glucose in the liver *via* gluconeogenesis (Huntington, 1990), and less methane (Thornton & Owens, 1981), which is not only a potent greenhouse gas but represents a gross energy loss of 2%–12% for the animal (Johnson & Johnson, 1995). Monensin treatment also reduces protein degradation in the rumen (Whetstone, Davis, & Bryant, 1981), allowing more feed protein to reach the abomasum (Falkner, Klopfenstein, Trotter, & Britton, 1985), where it is converted to amino acids that are absorbed in the intestine. In addition, monensin reduces the incidence of digestive disorders such as bloat (Bartley et al., 1983), acidosis (Dennis & Nagaraja, 1981), and ketosis (Sauer, Kramer, & Cantwell, 1989) and in dairy cows increases yields of milk and milk protein (McGuffey, Richardson, & Wilkinson, 2001).

Monensin's ability to effect these changes is due to its ability to alter the microbial populations within the rumen. Monensin achieves this by inserting itself into susceptible cell membranes where it acts as a metal/proton antiporter (Pressman, 1976), eliminating the H^+^, Na^+^, and K^+^ ion gradients across the cell membrane, resulting in the collapse of the proton motive force and eventually cell death (Russell, 1987; Russell & Strobel, 1989). Monensin is most effective against gram‐positive bacteria, because they lack the protective outer membrane of gram‐negative cells (Russell & Strobel, 1988). Thus, animals fed monensin have higher levels of gram‐negative bacteria that are more likely to produce propionate; and reduced levels of gram‐positive bacteria, that are more likely to degrade dietary protein (Russell & Strobel, 1988) and supply methanogens with hydrogen and formate for methanogenesis (Russell & Strobel, 1989). However, studies have shown that monensin supplementation does not always produce these effects (Hamilton, DePeters, McGarvey, Lathrop, & Mitloehner, 2010; Hook, Northwood, Wright, & McBride, 2009; McGarvey, Hamilton, DePeters, & Mitloehner, 2010; Odongo et al., 2007). Several studies have suggested that monensin's ability to alter the microbial populations within the rumen is dependent on both animal diet (Grainger, Williams, Eckard, & Hannah, 2010; Guan, Wittenberg, Ominski, & Krause, 2006) and monensin dosage (Duffield, Merrill, & Bagg, 2012; Ellis et al., 2012). However, there have not been any studies to date that have examined the dose‐dependent effects of monensin on the rumen microbiota of lactating dairy cows. We hypothesized that we could identify a dosage of monensin that is effective at altering the bacterial population structure of the rumen and thus reduce the levels of methanogenic archaea by feeding lactating cows a standardized diet supplemented with increasing levels of monensin and measuring the changes in the rumen microbiota. In this study, we fed lactating dairy cattle a standardized diet supplemented with three different dosages of monensin and monitored the changes in their rumen microbiota *via *16S gene sequence analysis and qPCR.

## 2 MATERIALS AND METHODS

### 2.1 Animals used in the study

Twelve multiparous, lactating Holstein dairy cows from the University of California, Davis dairy herd were used in this study. The animals were housed in a facility approved by the Association for Assessment and Accreditation of Laboratory Animal Care and all protocols were approved by the UC Davis Institutional Animal Care and Use Committee. The 12 animals were randomly assigned into four groups (three cows per group) and were fed the same basal total mixed ration diet, twice daily, that was top‐dressed with monensin at each respective dose: Control (0.0 mg cow^−1^ day^−1^), Low (175 mg cow^−1^ day^−1^), Medium (368 mg cow^−1^ day^−1^), or High (518 mg cow^−1^ day^−1^) for 20 days. The nutritional composition of the diet (Table 1) was analyzed by Cumberland Valley Analytical, Inc. (Hagerstown, MD, USA), and the concentration of monensin was determined by Covance Laboratories (Greenfield, IN).

**Table 1:** Composition of diet

Ingredient	% Total
Corn silage	36
Alfalfa hay	18
Whole cottonseed	9
Almond hulls	4
Grain mix^a^	33

Composition	(g kg^−1^)
Neutral detergent fiber	376
Acid detergent fiber	267
Starch	183
Crude protein	156
Ash	74
Sugar	56
Lignin	53
Crude fat	52
K	16
Non fiber carbohydrate	9
Ca	4
Mg	4
S	3
Na	3
Fe (mg kg^−1^)	332
Mn (mg kg^−1^)	87
Zn (mg kg^−1^)	76
Cu (mg kg^−1^)	22

a Grain Mix: 22% ground corn;18.5% ground wheat; 18.6% soybean hulls; 13.3% corn germ; 8.6% canola meal; 6.1% feather meal; 3.1% megalac; 2% CaCO3; 2% blood meal; 1.6% Na3H(CO3)2; 1.6% NaCl; 1.4% urea; 0.6% MgO; 0.3% PO4; and 0.6% vitamin & mineral premix (Cargill, Minneapolis, MN).

### 2.2 Rumen sampling

Rumen samples were collected from each cow before and after 20 days of monensin treatment to ensure the monensin treatment had enough time to take effect. Rumen fluid was collected from each animal *via* an oral stomach tube as described by Lodge‐Ivey, Browne‐Silva, and Horvath (2009). Briefly, approximately 2.5 hr after the feeding, cows were placed in a chute and a steel bovine mouth speculum was placed in the animal's mouth. A plastic stomach tube (0.6 cm I.D. and 3 m length) was inserted through the speculum into the rumo‐reticulum and approximately 250 ml of fluid was collected from each animal and transferred into 50 ml tubes (Becton Dickenson, Franklin Lakes, NJ, USA) that were immediately sealed, placed on ice and transported to the laboratory for analysis. Precautions were taken to prevent saliva contamination; however, it is possible that some contamination occurred.

### 2.3 16S rRNA gene library construction

DNA was extracted from 15 ml of rumen fluid as described by Yu and Morrison (2004). PCR amplification of 16S rRNA genes was carried out using the primers 27f (5′ AGAGTTTGATCCTGGCTCAG 3′) and 1392r (5′ GACGGGCGGTGTGTAC 3′) (Lane, 1991). PCR was performed as recommended by Polz and Cavanaugh (1998) to reduce bias in amplification. Briefly, 50 µl reactions contained 25 µl High Fidelity PCR Master Mix (Roche, Nutley, NJ, USA), 50 ng DNA and 1 µmole L^−1^ of each primer. PCR was performed in a Tetrad Thermocycler (Bio‐Rad, Hercules, CA, USA) under the following conditions: one cycle of 95°C for 5 min, 20 cycles of 94°C for 30 s, 55°C for 30 s, 72°C for 1.5 min, and one cycle of 10 min at 72°C. The PCR products were visually examined *via *agarose gel electrophoresis to ensure a single 13.5 Kbp band was produced, purified using the Zymo DNA Clean and Concentrator Kit (Zymo Research, Orange, CA, USA), cloned using the TOPO TA Cloning Kit (Invitrogen, Carlsbad, CA, USA) and transformed into *E. coli* TOP10 competent cells (Invitrogen). Clones were grown on LB agar (Fisher Scientific, Fair Lawn, NJ, USA) plates containing kanamycin (Km) (50 µg ml^−1^) at 37°C for 18 hr. Colonies were transferred to 96‐well plates with LB Km broth (Fisher Scientific). For each treatment, three animals were sampled for rumen fluid that was extracted for DNA. For each DNA sample, two 96‐well plates of sequences were analyzed, for a total of 576 sequences for each treatment group, and 2,304 sequences in total.

### 2.4 DNA template preparation and sequencing

DNA templates were prepared from 0.2 µl overnight cultures using the TempliPhi HT Amplification Kit (GE Healthcare, Piscataway, NJ, USA). Sequencing reactions were performed using the primer 1392r and the BigDye Terminator v3.1 Cycle Sequencing Kit (Applied Biosystems, Foster City, CA, USA). Sequencing reactions were purified using the BigDye XTerminator Purification Kit (Applied Biosystems); electrophoresis and readout were performed using an Applied Biosystems 3730XL Genetic Analyzer (Applied Biosystems).

### 2.5 DNA sequence and statistical analysis

DNA sequences were edited manually to correct falsely called bases and trimmed at both the 5′ and 3′ ends using the Seqman software (DNASTAR Inc., Madison, WI, USA) and analyzed for chimeras using UCHIME (Edgar, Hass, Clemente, Quince, & Knight, 2011). Sequences with reads of 600 bp covering hypervariable regions V5‐V8 were grouped into operational taxonomic units (OTU) (>97% sequence identity) using the FastGroup (Sequritan & Rohwer, 2001). Each OTU was assigned to a phylum using the Classifier software (Wang, Garrity, Tiedje, & Cole, 2007), available at the Ribosomal Database Project II (http://rdp.cme.msu.edu/index.jsp). Once classified, pair‐wise comparisons of the OTU were performed using the Ribosomal Database Project II Library Compare software (Wang et al., 2007). Sequences of all OTU were deposited in GenBank under submission SUB2986750. Simpson entropy, Chao1 estimates, and evenness were calculated using Estimate S (Colwell, 2006).

### 2.6 Quantitative PCR

Quantitative polymerase chain reaction (qPCR) for the methanogenic archaea DNA in rumen fluid was performed as described by Ohene‐Adjei et al. (2008) and for protozoa, it was as described by Sylvester, Karnati, Yu, Morrison, and Firkins (2004). For both experiments, standard curves of template DNA (methanogen or protozoan) were made to contain 1.0 × 10^8^–1 × 10^5^ copies per µl in 10‐fold serial dilutions. All qPCR was performed in triplicate using an Applied Biosystems 7500 Real‐Time PCR System (Life Technologies, Carlsbad, CA, USA). Significance of the qPCR was determined by the Student's *t* test with *p* < 0.05. Primers used for archaea were MB1174F GAGGAAGGAGTGGACGACG GTA and Arch1406R ACGGGCGGTGTGTGCAAG (Ohene‐Adjei et al., 2008); primers used for protozoa were 316f GCTTTCGWTGGTAGTGTATT and 539r CTTGCCCTCYAATCGTWCT (Sylvester et al., 2004). Efficiency of PCR was calculated by the equation *E* = −1 + 10^(−1/slope)^ and ranged between 89% and 108%.

## 3 RESULTS AND DISCUSSION

### 3.1 16S rRNA analysis

To determine the effects of monensin on the bacterial populations within the rumen we constructed 16S rRNA gene libraries from DNA extracted from rumen fluid of cows fed an identical diet (Table 1) supplemented with either no, low (175 mg cow^‐1^ day^‐1^), medium (368 mg cow^‐1^ day^‐1^), or high (518 mg cow^‐1^ day^‐1^) doses of monensin for 20 days (Table 2). The library derived from the rumen fluid of the cows fed the control diet (i.e., no monensin) contained sequences representative of 10 phyla (Table 2). The majority of these sequences were associated with the phyla *Bacteroidetes* (58.9%), *Firmicutes* (31.4%), and *Proteobacteria* (3.6%). These results are similar to previous studies that examined 16S rRNA genes libraries derived from lactating dairy cows (Kasparovska et al., 2016; Pitta et al., 2016). The library derived from the cows fed the low dose of monensin had no significant change (*p* > 0.05) in the percentage of sequences associated with any of the phyla. The library derived from the cows fed the medium dose had a significant increase in sequences associated with the phylum *Bacteroidetes* and a significant decrease in those associated with the *Firmicutes* (*p* < 0.05). The library derived from the cows fed the high dose of monensin contained a significant increase in sequences associated with the phyla *Bacteroidetes* and *Proteobacteria* and significant decrease in those associated with the phylum *Firmicutes*.

**Table 2:** Percentage of 16S rRNA gene sequences associated with bacterial phyla

Phylum	Monensin dosage			
	Control	Low	Medium	High
*Actinobacteria*	0.0	0.2	0.2	0.2
*Bacteroidetes*	58.9	62.8	65.8*	65.9*
Ca. Saccharibacteria	0.2	0.0	0.4	0.6
*Chloroflexi*	0.2	0.0	0.2	0.0
*Elusimicrobia*	0.0	0.0	0.0	0.2
*Firmicutes*	31.4	26.8	22.5*	19.0*
*Fibrobacteres*	1.3	1.8	0.6	0.4
*Lentisphaerae*	0.2	0.0	0.0	0.0
*Planctomycetes*	0.0	0.2	0.0	0.0
*Proteobacteria*	3.6	4.2	4.7	7.1*
*Spirochaetes*	1.7	0.4	1.3	1.8
*Synergistetes*	0.0	0.0	0.2	0.2
*Tenericutes*	0.4	0.6	0.4	0.2
TM7	0.4	0.0	0.4	0.6
*Verrucomicrobia*	0.0	0.0	0.2	0.6
Unclassified	2.1	3.2	3.4	3.8

^*^Significant difference from control library (*p* < 0.05)

These dose‐dependent changes in the bacterial populations are consistent with in vitro studies that showed the antibacterial activity of monensin is most effective against gram‐positive bacteria (Chow & Russell, 1990; Russell, 1987). While monensin is known to attach equally well to both gram‐positive and gram‐negative bacteria (Chow, Kessel, & Russell, 1994), its effectiveness against the gram‐positive bacteria is believed to be due to their lack of an outer membrane (Russell & Strobel, 1988). However this may be an oversimplification, as some gram‐positive bacteria have been shown to become adapted to monensin in vitro (Simjee, Heffron, Pridmore, & Shryock, 2012; Weimer, Stevenson, Mertens, & Hall, 2011) as‐well‐as in vivo (Weimer, Stevenson, Mertens, & Thomas, 2008). The adaptation of gram‐positive bacteria to monensin exposure has been associated with alterations in protein production, cell wall structure (Simjee et al., 2012) and the production of extracellular polysaccharides (Rychlik & Russell, 2002; Weimer et al., 2008). However, there are no resistance genes associated with this phenotype, and it is rapidly lost when selective pressure is removed (Simjee et al., 2012), suggesting it is epigenetic.

Monensin treatment also resulted in changes in the bacterial diversity within the rumen (Table 3). Feeding all doses of monensin reduced the number of operational taxonomic units (OTU), as estimated by Chao1 analysis, by approximately 25%. The Simpson indices, which incorporate species richness (or in this case OTU richness) and evenness, were also affected by the addition of monensin to the diet. At the low dose an increase in these indices was observed, and as the doses increased these indices decreased in a dose‐dependent manner. This effect on the microbial diversity in the gut of animals fed antibiotics has been observed previously (Looft et al., 2012; Suchodolski et al., 2009).

**Table 3:** Diversity statistics

Library (dosage)	No. of clones	Richness (No. OTU)	Chao1 estimate	Simpson Index (1/D)	Evenness Index (*E*)
Control	518	311	1,008	180	0.942
Low	492	302	755	268	0.957
Medium	500	313	761	252	0.956
High	502	311	755	167	0.940

### 3.2 Methanogenic archaea qPCR

Monensin has been shown to have little or no direct effect against methanogenic archaea (Russell & Houlihan, 2003); however, the decreases in gram‐positive bacteria within the rumen has been shown to reduce the concentration of methanogenic substrates (i.e., hydrogen and formate) needed by these microorganisms (Haney & Hoehn, 1968; Russell & Strobel, 1989). To determine the effect of monensin on the number of methanogens in the rumen, we performed qPCR on DNA extracted from the rumen fluid (Table 4). In the control group, no significant change was observed in methanogen sequences present after 20 days. However, significant decreases were observed for all levels of monensin treatment (*p* < 0.01). At the low and medium dose, we observed a 3.9‐ and 7.5‐fold decrease in these sequences, respectively. Interestingly, the high dose only decreased these sequences by ~twofold. These data are consistent with those of Hook et al. (2009), who observed a decrease in the number of methanogen sequences in rumen fluid after 20 days of monensin treatment. However, after 90 days of treatment, the methanogens recovered and no significant effect was observed over their 180‐day experiment. Likewise, Guan et al. (2006) reported a significant decrease in methane production after short‐term monensin treatment; however, normal methane production resumed after 4 weeks, leading the authors to speculate that the rumen bacteria had adapted to monensin.

**Table 4:** Methanogen 16S rRNA gene copies ng^−1^ DNA before and after monensin treatment

Animal group	Before (*SD*)	After (*SD*)	*p*‐Value
Control	3.71 × 10^6^ (4.37 × 10^5^)	3.02 × 10^6^ (2.43 × 10^6^)	0.420
Low	5.13 × 10^6^ (2.06 × 10^6^)	1.32 × 10^6^ (8.64 × 10^5^)	0.0001
Medium	4.13 × 10^6 ^(1.19 × 10^6^)	5.49 × 10^5^ (1.90 × 10^5^)	0.0001
High	5.42 × 10^6 ^(2.35 × 10^6^)	2.76 × 10^6^ (1.01 × 10^6^)	0.0075

Note

*SD*: standard deviation.

### 3.3 Protozoa qPCR

It is estimated that up to 25% of rumen methanogens are associated with protozoa (Newbold, Lassalas, & Jouany, 1995) that supply them with H_2_ and CO_2_ via their hydrogenosomes (Embley, Giezen, Horner, Dyal, & Foster, 2003) that they convert to methane, water, and energy (Wolin, 1974). To determine if the decreases in methanogens were related to the anti‐protozoan activity of monensin, we performed qPCR to quantify the number of protozoan 18S rRNA genes extracted from rumen fluid before and after monensin treatment. Our results showed that monensin had no significant effect on the number of protozoan 18S rRNA genes in the rumen fluid at any dosage tested (Table 5).

**Table 5:** Protozoan 18S rRNA gene copies ng^−1^ DNA before and after monensin treatment

Animal group	Before (*SD*)	After (*SD*)	*p*‐Value
Control	1.24 × 10^7^ (8.32 × 10^6^)	1.71 × 10^7 ^(2.69 × 10^6^)	0.75
Low	6.68 × 10^6^ (2.46 × 10^6^)	2.91 × 10^6^ (3.68 × 10^6^)	0.12
Medium	5.68 × 10^6^ (2.83 × 10^6^)	9.03 × 10^6^ (9.81 × 10^6^)	0.54
High	1.38 × 10^7^ (6.51 × 10^6^)	8.34 × 10^6^ (1.88 × 10^6^)	0.33

Note

*SD*: standard deviation.

Overall, the high dosage of monensin produced the greatest increase in the gram‐negative phyla *Bacteroidetes *and *Proteobacteria* and the greatest decrease in the gram‐negative phylum *Firmicutes*. However, the middle dosage also produced significant alterations in the phyla *Bacteroidetes* and *Firmicutes* and was more effective at reducing the methanogenic archaea than the high dosage. From these data, we conclude that for lactating dairy cows fed this diet the middle dosage was the most efficacious. Future studies are needed to examine the effects of these dosages on milk production and animal health to determine the economic return of using these dosages with this diet and to examine the possibility of bacterial adaptation to monensin over time.

## CONFLICT OF INTEREST

No conflict of interest declared.

## AUTHORS CONTRIBUTION

JM, SP, JP, and RN carried out bench work, data analysis and were involved in the manuscript preparation. SP and FM designed the study and performed all work related to sampling and care of the animals. All authors have read and approved the final manuscript.

## ETHICS STATEMENT

The animals were housed in a facility approved by the Association for Assessment and Accreditation of Laboratory Animal Care and all protocols were approved by the UC Davis Institutional Animal Care and Use Committee.

## Data Availability

All sequences were deposited in GenBank and are available under submission SUB4783994 accession numbers MK161521‐MK163307.

## References

[mbo3783-bib-0001] Bartley, E. E. , Nagaraja, T. G. , Pressman, E. S. , Dayton, A. D. , Katz, M. P. , & Fina, L. R. (1983). Effects of lasalocid or monensin on legume or grain (feedlot) bloat. Journal of Animal Science, 56, 1400–1406. 6874619 10.2527/jas1983.5661400x

[mbo3783-bib-0002] Chow, J. M. , & Russell, J. B. (1990). Effect of ionophores and pH on growth of *Streptococcus bovis* in batch and continuous culture. Applied and Environment Microbiology, 56, 1588–1593. 10.1128/aem.56.6.1588-1593.1990 PMC184476 2383003

[mbo3783-bib-0003] Chow, J. M. , Van Kessel, J. A. , & Russell, J. B. (1994). Binding of radiolabels monensin and lasalocid to ruminal microorganisms and feed. Journal of Animal Science, 72, 1630–1635. 8071190 10.2527/1994.7261630x

[mbo3783-bib-0004] Colwell, R. K. (2006). *Estimate S: Statistical estimation of species richness and shared species from samples. Version 8* . Retrieved from purl.oclc.org/estimates

[mbo3783-bib-0005] Dennis, S. M. , & Nagaraja, T. G. (1981). Effect of lasalocid or monensin on lactate‐producing or using rumen bacteria. Journal of Animal Science, 52, 418–426. 10.2527/jas1981.522418x 7275867

[mbo3783-bib-0006] Dinius, D. A. , Simpson, M. E. , & Marsh, P. B. (1976). Effect of monensin fed with forage on digestion and the ruminal ecosystem of steers. Journal of Animal Science, 42, 229–234. 10.2527/jas1976.421229x 2571

[mbo3783-bib-0007] Duffield, T. F. , Merrill, J. K. , & Bagg, R. N. (2012). Meta‐analysis of the effects of monensin in beef cattle on feed efficiency, body weight gain, and dry matter intake. Journal of Animal Science, 90, 4583–4592. 22859759 10.2527/jas.2011-5018

[mbo3783-bib-0008] Edgar, R. C. , Hass, B. J. , Clemente, J. C. , Quince, C. , & Knight, R. (2011). UCHIME improves sensitivity and speed of chimera detection. Bioinformatics, 27, 2194–2200. 10.1093/bioinformatics/btr381 21700674 PMC3150044

[mbo3783-bib-0009] Ellis, J. L. , Dijkstra, J. , Bannink, A. , Kebreab, E. , Hook, S. E. , Archibeque, S. , & France, J. (2012). Quantifying the effects of monensin dose on the rumen volatile fatty acid profile in high‐grain‐fed beef cattle. Journal of Animal Science, 90, 2717–2726. 22896736 10.2527/jas.2011-3966

[mbo3783-bib-0010] Embley, T. M. , Van Der Giezen, M. , Horner, D. S. , Dyal, P. L. , & Foster, P. (2003). Mitochondria and hydrogenosomes are two forms of the same fundamental organelle. Philosophical Transactions of the Royal Society B: Biological Sciences, 358, 191–203. 10.1098/rstb.2002.1190 PMC1693103 12594927

[mbo3783-bib-0011] Falkner, D. B. , Klopfenstein, T. J. , Trotter, T. N. , & Britton, R. A. (1985). Monensin effects on digestibility, rumen protein escape and microbial protein synthesis on high fiber diets. Journal of Animal Science, 61, 654–660. 2999053 10.2527/jas1985.613654x

[mbo3783-bib-0012] Grainger, C. , Williams, R. , Eckard, R. J. , & Hannah, M. C. (2010). A high dose of monensin does not reduce methane emissions of dairy cows offered pasture supplemented with grain. Journal of Dairy Science, 93, 5300–5308. 10.3168/jds.2010-3154 20965346

[mbo3783-bib-0013] Guan, H. , Wittenberg, K. M. , Ominski, K. H. , & Krause, D. O. (2006). Efficacy of ionophores in cattle diets for mitigation of enteric methane. Journal of Animal Science, 84, 1896–1906. 16775074 10.2527/jas.2005-652

[mbo3783-bib-0014] Hamilton, S. W. , DePeters, E. J. , McGarvey, J. A. , Lathrop, J. , & Mitloehner, F. M. (2010). Greenhouse gas, animal performance, and bacterial population structure responses to dietary monensin fed to dairy cows. Journal of Environmental Quality, 39, 106–114. 10.2134/jeq2009.0035 20048298

[mbo3783-bib-0015] Haney, M. E. , & Hoehn, M. M. (1968). Antimicrobial agents and chemotherapy‐1967 (p. 349). Ann Arbor, MI: American Society for Microbiology. 10.1128/AAC.7.3.349 5596158

[mbo3783-bib-0016] Hook, S. E. , Northwood, K. S. , Wright, A. D. G. , & McBride, B. W. (2009). Long‐term monensin supplementation does not significantly affect the quantity or diversity of methanogens in the rumen of the lactating dairy cow. Applied and Environment Microbiology, 75, 374–380. 10.1128/AEM.01672-08 PMC2620707 19028912

[mbo3783-bib-0017] Huntington, G. B. (1990). Energy metabolism in the digestive tract and liver of cattle: Influence of physiological state and nutrition. Reproduction Nutrition Développement, 30, 35–47. 10.1051/rnd:19900103 2184823

[mbo3783-bib-0018] Johnson, K. A. , & Johnson, D. E. (1995). Methane emissions from cattle. Journal of Animal Science, 73, 2483–2492. 10.2527/1995.7382483x 8567486

[mbo3783-bib-0019] Kasparovska, J. , Pecinkova, M. , Dadakova, K. , Krizova, L. , Hadrova, S. , Lexa, M. , … Kasparovsky, T. (2016). Effects of isoflavone‐enriched feed on the rumen microbiota in dairy cows. PLoS One, 11(4), e0154642 10.1371/journal.pone.0154642 27124615 PMC4849651

[mbo3783-bib-0020] Lane, D. J. (1991). 16S/23S rRNA sequencing In Stakebrandt E. & Goodfellow M. (Eds.), Nucleic acid sequencing techniques in bacterial systematic (pp. 115–175). New York, NY: Wiley and Sons.

[mbo3783-bib-0021] Lodge‐Ivey, S. L. , Browne‐Silva, J. , & Horvath, M. B. (2009). Bacterial diversity and fermentation end products in rumen fluid samples collected via oral lavage or rumen cannula. Journal of Animal Science, 87, 2333–2337. 19329475 10.2527/jas.2008-1472

[mbo3783-bib-0022] Looft, T. , Johnson, T. A. , Allen, H. K. , Bayles, D. O. , Alt, D. P. , Stedtfeld, R. D. , … Stanton, T. B. (2012). In‐feed antibiotic effects on the swine intestinal microbiome. Proceedings of the National Academy of Sciences of the United States of America, 109, 1691–1696. 10.1073/pnas.1120238109 22307632 PMC3277147

[mbo3783-bib-0023] McGarvey, J. A. , Hamilton, S. W. , DePeters, E. J. , & Mitloehner, F. (2010). Effect of dietary monensin on the bacterial population structure of dairy cattle colonic contents. Applied Microbiology and Biotechnology, 85, 1947–1952. 10.1007/s00253-009-2229-8 19784643

[mbo3783-bib-0024] McGuffey, R. K. , Richardson, L. , & Wilkinson, J. I. D. (2001). Ionophores for dairy cattle: Current status and future outlook. Journal of Dairy Science, 84(E. Suppl.), E194–E203. 10.3168/jds.S0022-0302(01)70218-4

[mbo3783-bib-0025] Newbold, C. J. , Lassalas, B. , & Jouany, J. P. (1995). The importance of methanogens associated with ciliate protozoa in ruminal methane production in vitro. Letters in Applied Microbiology, 21, 230–234. 10.1111/j.1472-765X.1995.tb01048.x 7576513

[mbo3783-bib-0026] Odongo, N. E. , Bagg, R. , Vessie, G. , Dick, P. , Or‐Rashid, M. M. , Hook, S. E. , … McBride, B. W. (2007). Long‐term effects of feeding monensin n methane production in lactating dairy cows. Journal of Dairy Science, 90, 1781–1788. 17369219 10.3168/jds.2006-708

[mbo3783-bib-0027] Ohene‐Adjei, S. , Chaves, A. V. , McAllister, T. A. , Benchaar, C. , Teather, R. M. , & Forster, R. J. (2008). Evidence of increased diversity of methanogenic archaea with plant extract supplementation. Microbial Ecology, 56, 234–242. 10.1007/s00248-007-9340-0 18075710

[mbo3783-bib-0028] Pitta, D. W. , Indugu, N. , Kumar, S. , Vecchiarelli, B. , Sinha, R. , Baker, L. D. , … Ferguson, J. D. (2016). Metagenomic assessment of the functional potential of the rumen microbiome in Holstein dairy cows. Anaerobe, 38, 50–60. 10.1016/j.anaerobe.2015.12.003 26700882

[mbo3783-bib-0029] Polz, M. F. , & Cavanaugh, C. M. (1998). Bias in template‐to‐product ratios in multitemplate PCR. Applied and Environment Microbiology, 64, 3724–3730. 10.1128/aem.64.10.3724-3730.1998 PMC106531 9758791

[mbo3783-bib-0030] Pressman, B. C. (1976). Biological applications of ionophores. Annual Review of Biochemistry, 45, 501–503. 10.1146/annurev.bi.45.070176.002441 786156

[mbo3783-bib-0031] Richardson, L. F. , Raun, A. P. , Potter, E. L. , Cooley, C. O. , & Rathmacher, R. P. (1976). Effect of monensin on rumen fermentation in vitro and in vivo. Journal of Animal Science, 43, 657–664.

[mbo3783-bib-0032] Russell, J. B. (1987). A proposed mechanism of monensin action in inhibiting ruminal bacterial growth: Effects on ion flux and protonmotive force. Journal of Animal Science, 64, 1519–1525. 3583956 10.2527/jas1987.6451519x

[mbo3783-bib-0033] Russell, J. B. , & Houlihan, A. J. (2003). Ionophore resistance of ruminal bacteria and its potential impact on human health. FEMS Microbiology Reviews, 27, 65–74. 10.1016/S0168-6445(03)00019-6 12697342

[mbo3783-bib-0034] Russell, J. B. , & Strobel, H. J. (1988). Effects of additives on in vitro ruminal fermentation: A comparison of monensin and bacitracin, another Gram‐positive antibiotic. Journal of Animal Science, 66, 552–558. 10.2527/jas1988.662552x 3372392

[mbo3783-bib-0035] Russell, J. B. , & Strobel, H. J. (1989). Effect of ionophores ruminal fermentation. Applied and Environment Microbiology, 55, 1–6. 10.1128/aem.55.1.1-6.1989 PMC184044 2650616

[mbo3783-bib-0036] Rychlik, J. L. , & Russell, J. B. (2002). The adaptation and resistance of *Clostridium aminophilum* F to the *Butyrivibrio fibrisolvens *JL5 and monensin. FEMS Microbiology Letters, 209, 93–98. 12007660 10.1111/j.1574-6968.2002.tb11115.x

[mbo3783-bib-0037] Sauer, F. D. , Kramer, J. K. G. , & Cantwell, W. J. (1989). Antiketogenic effects of monensin in early lactation. Journal of Dairy Science, 72, 436–442. 10.3168/jds.S0022-0302(89)79125-6 2703566

[mbo3783-bib-0038] Sequritan, V. , & Rohwer, F. (2001). FastGroup: A program to dereplicate libraries of 16S rDNA sequences. BMC Bioinformatics, 2, 9. 11707150 10.1186/1471-2105-2-9 PMC59723

[mbo3783-bib-0039] Simjee, S. , Heffron, A. , Pridmore, A. , & Shryock, T. R. (2012). Reversible monensin adaptation in *Enterococcus faecium*, *Enterococcus faecalis* and *Clostridium perfringens* of cattle origin: Potential impact on human food safety. Journal of Antimicrobial Chemotherapy, 67, 2388–2395. 10.1093/jac/dks236 22740589

[mbo3783-bib-0040] Suchodolski, J. S. , Dowd, S. E. , Westermarck, E. , Steiner, J. M. , Wolcott, R. D. , Spillmann, T. , & Harmoinen, J. A. (2009). The effect of the macrolide antibiotic tylosin on microbial diversity in the canine small intestine as demonstrated by massive parallel 16S rRNA gene sequencing. BMC Microbiology, 9, 210 10.1186/1471-2180-9-210 19799792 PMC2759960

[mbo3783-bib-0041] Sylvester, J. T. , Karnati, S. K. R. , Yu, Z. , Morrison, M. , & Firkins, J. L. (2004). Development of an assay to quantify rumen ciliate protozoal biomass in cows using real‐time PCR. Journal of Nutrition, 134, 3378–3384. 10.1093/jn/134.12.3378 15570040

[mbo3783-bib-0042] Thornton, J. H. , & Owens, F. N. (1981). Monensin supplementation and in vivo methane production by steers. Journal of Animal Science, 52, 628–634. 10.2527/jas1981.523628x 6267003

[mbo3783-bib-0043] Wang, Q. G. , Garrity, M. , Tiedje, J. M. , & Cole, J. R. (2007). Naïve Bayesian classifier for rapid assignment of rRNA sequences into the new bacterial taxonomy. Applied and Environment Microbiology, 73, 5261–5267. 10.1128/AEM.00062-07 PMC1950982 17586664

[mbo3783-bib-0044] Weimer, P. J. , Stevenson, D. M. , Mertens, D. R. , & Hall, M. B. (2011). Fiber digestion, VFA production, and microbial population changes during in vitro ruminal fermentations of mixed rations by monensin‐adapted and unadapted microbes. Animal Feed Science and Technology, 169, 68–78. 10.1016/j.anifeedsci.2011.06.002

[mbo3783-bib-0045] Weimer, P. J. , Stevenson, D. M. , Mertens, D. R. , & Thomas, E. E. (2008). Effect of monensin feeding and withdrawal on populations of individual bacterial species in the rumen of lactating dairy cows fed high‐starch rations. Applied Microbiology and Biotechnology, 80, 135–145. 10.1007/s00253-008-1528-9 18535825

[mbo3783-bib-0046] Whetstone, H. D. , Davis, C. L. , & Bryant, M. P. (1981). Effect of monensin on breakdown of protein by ruminal microorganisms in vitro. Journal of Animal Science, 53, 803–809. 7319956 10.2527/jas1981.533803x

[mbo3783-bib-0047] Wolin, M. J. (1974). Metabolic interactions among intestinal microorganisms. American Journal of Clinical Nutrition, 27, 1320–1328. 10.1093/ajcn/27.11.1320 4217102

[mbo3783-bib-0048] Yu, Z. , & Morrison, M. (2004). Improved extraction of PCR‐quality community DNA from digesta and fecal samples. BioTechniques, 36, 808–812. 10.2144/04365ST04 15152600

